# Albumin Is an Integrative Protein of Blood Plasma and Beyond

**DOI:** 10.3390/ijms252312627

**Published:** 2024-11-25

**Authors:** Daria A. Belinskaia, Richard O. Jenkins, Nikolay V. Goncharov

**Affiliations:** 1 Sechenov Institute of Evolutionary Physiology and Biochemistry of the Russian Academy of Sciences, 194223 St. Petersburg, Russia; d_belinskaya@mail.ru; 2 Leicester School of Allied Health Sciences, De Montfort University, The Gateway, Leicester LE1 9BH, UK; richard.jenkins@dmu.ac.uk

## 1. Albumin Structural Features

Albumin is a major protein in mammalian blood plasma or serum, where its concentration in healthy organisms is about 600 μM. The albumin molecule consists of three domains (I, II, and III), each of which includes two subdomains (A and B) [1]. The main endogenous ligands of albumin are fatty acids (FAs) [2], which can bind to seven fatty acid binding sites, FA1–7 [3,4]. Two additional FA binding sites (FA8 and FA9) are located in the cleft between domains I and III and can only be occupied in the presence of short-chain FAs or saturating amounts of FAs, respectively [4]. The binding of exogenous ligands occurs at two main drug sites: site 1 (Sudlow site I, overlaps with FA7 in subdomain IIA) and site 2 (Sudlow site II, overlaps with FA3&4 in subdomain IIIA) [4,5]. Site 3 (located in subdomain IB and overlapping with FA1), which binds compounds such as hemin and bilirubin, was later also identified [6,7]. Some ligands (e.g., thyroxine and lidocaine) can bind in the cleft between domains I and III [8,9]. The albumin molecule contains 17 disulfide bonds and a free thiol group of Cys34 [1].

## 2. Albumin Esterase Activity

In blood plasma, albumin maintains colloid osmotic pressure and performs a transport function [10]. In 1959, Casida and Augustinsson suggested that, in addition to its binding capacity, albumin possesses hydrolytic activity [11]. Since then, data have accumulated on the pseudoesterase (the irreversible covalent binding of a substrate to albumin with the subsequent release of only one of two products from the hydrolytic cleavage into a solution while the second remains in the adduct) and true esterase (the interaction of a substrate with the active center of albumin, with the formation of an enzyme–substrate complex and the subsequent disintegration of the complex into the enzyme and two products from the hydrolytic reaction) activity of albumin towards esters, including esters of phosphoric and phosphonic acids [12,13,14].

Tyr411 at site 2 is the major site of pseudoesterase activity in albumin [12]. Using NMR-spectroscopy, spectrophotometry, and molecular modeling, we analyzed the hydrolysis of nitrophenyl acetate (NPA) in the presence of albumin, which allowed us to establish that serum albumin has true esterase activity at site 1 with catalytic Tyr150 [15,16]. The kinetic constants of the (pseudo)esterase activity of human (HSA), bovine (BSA), and rat (RSA) albumins towards NPA and paraoxon (POX) indicate that the characteristics of albumin hydrolytic activity have species-specific differences, which are more pronounced for true esterase activity compared to pseudoesterase activity and less pronounced between HSA and RSA compared to BSA [17]. We proposed a scheme for describing the hydrolytic activity of albumin, which can be used to describe the functioning of both the sites of pseudo- and true esterase activity separately, as well as all hydrolytic activity of albumin at the two sites [16]. The structure of the small and virtually non-polar site contributes to the higher affinity of the site for the NPA and POX molecules and the stability of the enzyme–substrate complex compared to the capacious and polar site 1 [18]. This, in turn, promotes faster acetylation or phosphorylation of Tyr411 compared to Tyr150 at site 1, which is consistent with known experimental data and our proposed scheme. In our work [19], published in the presented Special Issue, we showed that the structure of site 1 promotes faster reactivation compared to site 2, which also indicates that site 1 can be classified as the site of true esterase activity of albumin.

## 3. Albumin Activity Modulation

It is known that allosteric modulation is a characteristic of the albumin molecule: ligand binding at one site affects binding and/or the rate of hydrolysis at another site [20]. We demonstrated that site 1 is more susceptible to allosteric modulation compared to site 2 [16,17]. On the example of BSA with the help of molecular modeling methods, we found that ligand binding at site 2 restricts the conformational mobility of domain III, and the formation of a hydrogen bond between the amino acids Asp493 and Thr495 plays a key role in this process. Due to the limited mobility of domain III, domain I changes its conformation to maintain contact with domain III, which affects the conformation of a ligand at site 1 [16].

The targeted modulation of albumin functional properties can solve many problems in toxicology and pharmacology. In rodents, we demonstrated the possibility of modulating albumin activity in acute organophosphate poisoning by reducing the affinity of the toxic compound for albumin and detoxifying the organophosphate molecules by blood plasma esterases [21]. In the paper by Deryusheva et al. [22], published in our Special Issue, a comprehensive search was performed for clinically approved drugs capable of changing the affinity of albumin for the β-amyloid peptide monomer (Aβ, a key pathogenic factor in the development of Alzheimer’s disease, AD). A promising approach to the prevention of AD is to reduce free Aβ levels by targeting the binding of Aβ to HSA. It has been found that prednisone favors HSA–Aβ interactions, mefenamic acid shows the opposite effect, and levothyroxine exhibits bidirectional effects. The obtained results provide a basis for drug repurposing for AD prevention and for the search of medications promoting AD progression [22].

## 4. Albumin Modification

In addition to the non-covalent binding of endogenous and exogenous ligands, albumin can be subject to covalent modifications. Thus, the free thiol group of Cys34 causes redox modifications of albumin: cysteinylation, homocysteinylation, and sulfinylation [23]. Another known type of albumin modification is its conjugation with sugars. Albumin differs from other blood proteins in that it is normally not glycosylated (not glycated, if we mean exclusively non-enzymatic glycosylation), although even a small percentage of glycated albumin makes a significant contribution to the pathogenesis of diabetes and other diseases. The most common glycation of albumin is with lysine and arginine residues [24].

Nitrosylation of the thiol group of Cys34 is another type of protein modification [25,26]. S-nitroso human serum albumin (SNO-HSA) has been suggested to serve in vivo as a reservoir for NO produced by endothelial cells [27]. The targeted modification of albumin may serve the purposes of translational medicine. In the paper by Mitin et al. [28], published in our Special Issue, with the aim of developing new effective and safe contrast agents for magnetic resonance imaging (MRI), a macromolecular construct based on HSA and nitroxyl radicals (HSA-NIT) was created using a new synthesis method that significantly increased the modification to 21 nitroxide residues per protein. HSA-NITs may represent a new class of MRI relaxation-based contrast agents as well as novel cleavable conjugates for use as hyperpolarizing contrast agents in MRI.

## 5. Albumin in Diagnostics

### 5.1. Albumin Molecules as a Diagnostic Biomarker

Due to the fact that albumin is the first to be affected in conditions of hyperglycemia and increased concentrations of reactive oxygen species (ROS), the level of modified albumin serves as a diagnostic and prognostic marker of various pathological conditions. Several papers in our Special Issue are devoted to research in this direction. Paar et al. [29] aimed to perform a comparative analysis of the level of reduced (HMA), reversibly (HNA1), and irreversibly (HNA2) oxidized albumin in patients with type 1 (T1DM) and type 2 (T2DM) diabetes mellitus and to determine the relationship between these indices and the age of patients, the duration of diabetes, and glycemic control. It was found that HNA2 was positively correlated with diabetes duration, which supports the hypothesis that oxidative stress plays an important role in DM pathogenesis. The level of oxidized HSA was increased in people with an increased glycated hemoglobin, which emphasizes the importance of diabetes control on systemic oxidative burden.

Wybranowski et al. measured the mean fluorescence lifetime (mFLT) of blood and plasma from healthy subjects and patients with COVID-19 pneumonia during illness and after recovery, as well as the mFLT of native and chloramine-T-oxidized albumin [30]. A significant decrease in blood and plasma mFLT was found in COVID-19 patients compared to healthy people and the patients after recovery. For both blood and plasma, mFLT was found to be negatively correlated with several severity biomarkers and advanced oxidation protein products, while it was positively correlated with albumin and the albumin-to-globulin ratio. The obtained result indicates that the decrease in mFLT of the blood and plasma of patients is associated with the oxidation of albumin caused by oxidative stress.

Klinkmann et al. [31] analyzed the blood of intensive care patients, dividing them into three groups: patients without visible signs of sepsis, patients with sepsis, and patients with septic shock. The subjects were measured for albumin binding capacity (ABiC), fatty acid binding efficiency (BE), and detoxification efficiency (DTE), as well as standard biochemical parameters. They found that ABiC, BE, and DTE effectively differentiated control patients from patients with sepsis or septic shock. Albumin functionality correlated with parameters of inflammation, liver failure, or kidney failure.

The review by Georgieva et al. [32], devoted to stable nitroxide as a diagnostic tool for monitoring oxidative stress and hypoalbuminemia, provides extensive information on the relationship between oxidative stress, inflammation, and low albumin levels, which are hallmarks of COVID-19. Briefly, low albumin levels are due to amino acid malabsorption, reduced albumin synthesis in hepatocytes, increased albumin excretion, leakage of albumin outside the vascular bed into the interstitial space, or combinations of these factors. In general, hypoalbuminemia reflects an increased rate of protein turnover resulting from an increased rate of catabolism. For example, the acute development of hypoalbuminemia in sepsis or trauma is due to increased capillary permeability to albumin, which leads to its redistribution from the vascular to interstitial spaces. High levels of reactive oxygen and nitrogen species generated during the acute phase of COVID-19 may lead to irreversible oxidative damage to albumin and impairment of its antioxidant properties, which in turn may lead to large-scale organ damage [32].

A study of elderly people with acute ischemic stroke (AIS), chronic cerebrovascular insufficiency (CCCI), prediabetes or newly diagnosed T2DM, or subcortical ischemic vascular dementia (SIVD) revealed the highest number of significant deviations from age-matched healthy donors (HDs) in the SIVD group, namely twenty, of which twelve were specific and six were non-specific, but with maximum differences from the HD group. The maximum decrease in albumin level is one of the most important non-specific indicators of the SIVD group [33]. Immunological and biochemical abnormalities in SIVD patients indicated persistent microvascular remodeling, chronic inflammation, and a significant reduction in the anabolic function of the liver and other tissues.

### 5.2. Albumin as a Component of Complex Biomarkers: Biochemical Basis of the Integrative Role of Albumin

In translational medicine, the relationship between oxidative stress, inflammation, and low native albumin levels is reflected in the inclusion of albumin concentration in a wide range of biomarkers of disease severity, described in our current and previous [34] Special Issues. Unlike individual biochemical or physiological indices, derivative indices allow for a better characterization of a complex syndrome as a set of specific symptoms and, therefore, a more rapid adoption of a set of adequate measures. In our paper [35], we reported the results of a pilot study of patients with COVID-19, which yielded a number of diagnostic indices characterizing the severity of the patient’s condition and the likelihood of death. Of particular interest is the new index [Urea] × [MDA] × 1000/(BChEb × [ALB]), which was 10 times higher in the group of deceased patients than in the group of survivors and 26 times higher than in the group of healthy elderly people. Expanding the patient cohort and the range of biochemical parameters to include the von Willebrand factor antigen [AGWF] allowed us to identify additional indices as the best predictors of COVID-19 infection-associated mortality, [Urea] × [AGWF] × 1000/(BChEb × [ALB]), followed by the much cheaper and more accessible to most clinics [Urea] × 1000/(BChEb × [ALB]) [36].

All these indices are characterized by the presence of the urea level in the numerator, butyrylcholinesterase (BChE) activity towards butyrylthiocholine (BChEb) and the albumin level. These parameters reflect the functional state of the liver, namely its protein-synthesizing function (albumin and BChE) and its generation of urea. This pattern is easily explained from the point of view of energy costs associated with anabolism and the neutralization of the main metabolic products—ammonia and carbon dioxide. Ammonia is a cytotoxic metabolite that is removed in humans and other mammals primarily by hepatic ureagenesis. Hyperammonemia occurs in advanced liver, heart, and lung diseases, as well as in cases of deficiency of urea cycle enzymes [37]. During ureagenesis, two amino groups, one from the free ammonium ion and the other from aspartate, as well as a carbon atom from the bicarbonate ion, are converted into a relatively non-toxic excretory product, urea. This occurs due to four “high-energy” phosphate bonds: three ATP molecules are hydrolyzed to two ADP molecules and one AMP molecule [38]. Two ATP molecules are required in the reaction of the formation of carbamoyl phosphate from ammonium and bicarbonate ions in mitochondria, catalyzed by carbamoyl phosphate synthetase I (CPS1). The breakdown of ATP to AMP and pyrophosphate occurs in the cytoplasm in a condensation reaction between the amino group of aspartate and the carbonyl group of citrulline to form argininosuccinate, catalyzed by argininosuccinate synthetase (ASS). The energy costs of hepatocytes associated with protein metabolism, the synthesis of amino acids and urea, and the work of Na+/K+-ATPase and transporters of Ca2+, account for approximately 60% of the total costs [39]. Moreover, of the 80% of oxygen consumption associated with ATP synthesis, up to 30% is due to protein synthesis but only 3% is due to ureagenesis [40]. Consequently, with increased catabolism, there is a potential for a sharp increase in energy consumption associated with ureagenesis, to the detriment of the protein-synthesizing function of the liver. In addition, it should be taken into account that during inflammatory processes, the need for the synthesis of proinflammatory cytokines and immunoglobulins increases [41], so that a decrease in the protein-synthesizing function of the liver is ensured by negative acute-phase proteins, primarily albumin.

Increased ammonia uptake and metabolism by skeletal muscle is the major mechanism of non-hepatic ammonia utilization. Non-hepatic utilization of ammonia occurs in the mitochondria through the synthesis of glutamate from α-ketoglutarate, leading to cataplerosis [37]. Ammonia also causes disruptions in the complex I function of the electron transport chain, with decreased NADH oxidation and electron leakage in complex III, and subsequent oxidative modifications of proteins (carbonylation) and lipids (lipid peroxidation). It is important to note that in hepatocytes, about 45% of ROS is generated in the endoplasmic reticulum and only 15% in mitochondria [42,43,44]. Both deficiency and excess of ROS lead to disruptions in oxidative protein folding and unfolded protein response (UPR)-induced neurodegeneration [44]. So, along with a decrease in the amount of albumin, a disruption in the structure of the synthesized protein is very likely, since the tertiary structure of one albumin molecule is largely determined by 17 (!) double bonds formed between the thiol residues of 34 cysteine residues as a result of oxidative folding.

The use of anaplerotic substrates to reverse ammonia-induced mitochondrial dysfunction is a novel therapeutic approach [37]. At the same time, we have established the effect of increasing the physical endurance of humans and experimental animals due to ammonium preconditioning with small doses of ammonium salts [45,46,47]. This effect appears to be due to the activation of CPS1, and hence the ornithine cycle, in periportal hepatocytes. This allows for saving on ATP energy directed towards the synthesis of glutamine in perivenous hepatocytes. The activation of CPS1 and the ornithine cycle is particularly pronounced when ammonium carbonate is used as a preconditioning agent, as compared with ammonium chloride [47]. Another effect of ammonium preconditioning is likely the inhibition of glutaminase, which catalyzes the generation of additional ammonia from glutamine in periportal hepatocytes, proximal tubular epithelium, endothelial cells, and lymphocytes. As a result, the capacity of the urea cycle increases without activating glutaminase and endogenous sources of carbon dioxide required for the activation of CPS1. This approach deserves further attention, as it allows for a reduction in the degree of cataplerotic reactions and the preservation of the protein-synthesizing function of the liver, in particular its ability to create physiologically complete albumin.

## 6. Albumin Interaction with Macromolecules

Albumin activity is not limited to interactions with low-molecular ligands entering the blood: albumin has the properties of a ligand for some receptors and enzymes [48]. In health, albumin is an endogenous inhibitor of angiotensin I-converting enzyme (ACE) and thus plays an important role in the blood pressure regulation system [49]. Additionally, albumin is not glycated. However, in a hyperglycemic environment, even a small percentage of glycated albumin (due to its high concentration) makes a significant contribution to the pathogenesis of diabetes mellitus and related diseases by binding to the receptor for advanced glycation end products (RAGE) [50]. In health, with an intact endothelium, albumin can leave the lumen of microvessels via transcytosis, and when microvessels are damaged, for example in a hyperglycemic environment, albumin can uncontrollably penetrate the blood–brain barrier, interact with receptors of proinflammatory cytokines, and trigger inflammatory cascades [51]. There is little information about the molecular mechanisms behind the interaction between albumin and macromolecules. Their study remains relevant, however, since such information can be useful for understanding the role of albumin in the pathogenesis of various diseases and can contribute to the development and improvement of therapy. In our computational experiments, we found that subdomains IB and IIIB of albumin play a key role in protein binding to a range of macromolecules under normal conditions, while in pathology, when albumin is modified, the sites and efficiency of interaction changes [52,53].

## 7. Conclusions

The results of the studies described in our two Special Issues attest to the integrative role of albumin, as a link between various tissues and organs. Albumin, which until recently was assigned a modest role as an osmotically active component, is essentially a molecular core, indicating and largely determining the health of the entire organism. Modern diagnostics, the pathogenesis of various diseases, and the development of therapeutic agents are currently unthinkable without a comprehensive consideration of the physicochemical, evolutionary–genetic, and physiological–biochemical characteristics of albumin.

## Data Availability

Data are contained within the article.
